# Laboratory evidence for the hematopoietic potential of *Beta vulgaris* leaf and stalk extract in a phenylhydrazine model of anemia

**DOI:** 10.1590/1414-431X20187722

**Published:** 2018-10-11

**Authors:** I. Gheith, A. El-Mahmoudy

**Affiliations:** 1 Department of Clinical Laboratory Sciences, Faculty of Applied Medical Sciences, Taibah University, Medinah, Saudi Arabia; 2 Department of Biotechnology, Animal Health Research Institute, Dokki, Egypt; 3 Department of Pharmacology, Benha University Faculty of Veterinary Medicine, Moshtohor, Egypt

**Keywords:** Anemia, Beta vulgaris, Beet, Hematinic, Anti-anemic, Antioxidant

## Abstract

This study was designed to provide laboratory evidence supporting the hematopoietic effect of *Beta vulgaris* (beet) leaf aqueous extract in phenylhydrazine-induced anemia model in albino rats. Extraction of the leaves/stalks was done by maceration in 30% hydro-ethanol for 48 h. An intraperitoneal injection of 20 mg/kg phenylhydrazine was applied for two consecutive days to develop hemolytic anemia on the 4th day after the 1st injection in 24 of 30 male albino rats. The animals were divided into 5 groups and received the following treatments: standard (ferrous ascorbate + folic acid; 13.5 + 0.135 mg/kg), *B. vulgaris* extract (100 and 200 mg/kg), or left untreated (normal and diseased controls). Blood samples were taken at 0, 4, 8, and 12 days of the experiment for hematological and clinico-chemical analysis. Beet leaf extract significantly restored the levels of red blood cells, white blood cells, hemoglobin, and hematocrit in dose- and time-dependent manners. Blood indices have been significantly corrected. Erythropoietin level was maintained at higher levels. Erythrocytic membrane oxidation biomarker (malondialdehyde) level was significantly reduced compared to the anemic untreated group. The extract exhibited potent, concentration (4–512 μg/mL)-dependent antioxidant activity indicated by the 2,2-diphenyl-1-picryl-hydrazyl (DPPH) assay, with IC50 value of 37.91 μg/mL. Beet leaf extract resulted in detection of flavonoid and phenolic compounds that may underlie its hematinic properties. These findings may indicate *B. vulgaris* as a good natural source for pharmaceutical preparations with hematopoietic effects and treatment of anemia and/or associated conditions.

## Introduction

Anemia is a common and major public health problem in several developing countries including Egypt and Saudi Arabia. Global estimates showed that 43% of children and 33% of non-pregnant women were anemic, with the highest incidence in Africa and South Asia (1). Regardless of categories and causes of anemia, it can be defined as a condition of having a less than normal quantity of hemoglobin and/or red blood cell (RBC) count. Such low levels of hemoglobin and RBCs may decrease the ability of the blood to distribute oxygen to different body organs, and thus, uncontrolled anemia may be serious or even life threatening (2). Among different anemias, the hemolytic one is a common class of anemia that may be inherited (due to deficiency of glucose-6-phosphate dehydrogenase) or acquired (due to exposure to hemolytic agents) with the result of intra- or extra-vascular destruction of RBCs (3). Exposure to some chemicals, including drugs may be associated with RBCs destruction along the course of therapy (4). Oxidative stress is also a predisposing factor and direct cause of various blood disorders by peroxidation of erythrocytic membranes (5).

Innovating and discovering therapeutic agents from safe sources has been receiving considerable attention of pharmacologists due to the crucial role that could be played by herbal medicine in prophylaxis and/or therapy of diseases, as well as improving the health status and performance of normal subjects (6).

*B. vulgaris* (also known as beet) is a plant belonging to the *Amaranthaceae* family (formerly placed in *Chenopodiaceae*), *Plantae* kingdom. It is distributed worldwide including subtropical and tropical countries in Africa and in Asia (7). The leaf, leaf stalks, and roots of beet plants are edible and may grow to 0.5–0.75 meters. The red/purple color of beetroot is due to a variety of betalain pigments, unlike most other red plants that contain anthocyanin pigments (8).

The foliage of the red beet is a delicious green vegetable, with higher contents of various nutrients than in the roots. Beet foliage is rich in carotenoids (beta carotene, lutein, and zeaxanthin), flavonoids, and vitamin C, which are strong antioxidants, and folic acid (a component of vitamin B complex, which is needed to release energy) and is useful in the functioning of the nervous and immune systems and in hematopoiesis (9).

*B. vulgaris* has been used in folk medicine and some of its pharmacological activities have been demonstrated, including vasodilating (10), antihypertensive (11,12), antidiabetic (13), hepatoprotective (14), and anticancer (15); it has also been shown to increase athletic performance (16).

The present study, therefore, aimed to apply *in vitro* and *in vivo* assays to investigate the possible favorable hematopoietic and anti-anemic potentials of a hydroethanolic extract of *B. vulgaris* leaves and stalks in a phenylhydrazine-induced anemia model, and to identify the active principle(s) contained in the extract that might mediate such potentials.

## Material and Methods

### Plant part used

**Figure 1.**
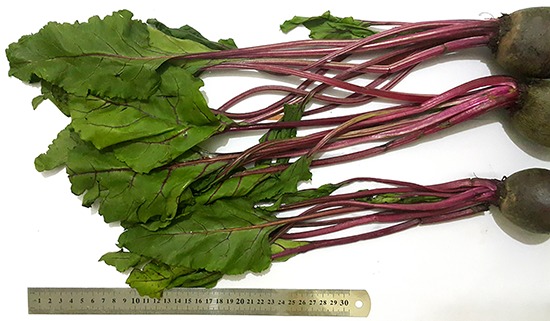
Photograph of the leaves and stalks of *Beta vulgaris* plant used for extraction.

The green parts of *Beta vulgaris* (Figure 1) were collected from our local environment (Qalioubeya Governorate, Egypt) and identified by a Botany specialist.

### Chemicals and reagents

Phenylhydrazine and DPPH^-^ (2,2-diphenyl-1-picryl-hydrazyl) were obtained from Sigma-Aldrich Chemical Co. (USA). The standard anti-anemic drug was obtained from Fefoli^®^, a patent suspension that is formulated as 100 mg of ferrous ascorbate +1.5 mg of folic acid/mL in 150 mL bottle, a product of Metlar^®^ Formulations (India). All other chemicals/solutions/reagents used were of analytical grade. Reagents used for detection of phenolic and flavonoid phytochemical groups were prepared in our laboratory.

### Extraction procedure

The adopted methodological procedures of extraction were modified according to Harborne (17). Plant green parts were refluxed in running tap water and then with bi-distilled water, shade-dried at room temperature and coarsely chopped using a clean knife and board. After drying, leaf and stalk extract was prepared by macerating a weighed amount (200 g) of the dried chopped parts in a known volume (2 L) of aqueous:organic solvent (distilled water:ethanol, 70:30, v/v) in stoppered Erlenmeyer flasks. Maceration continued for 48 h under refrigeration with occasional mixing. The hydro-ethanolic extract was filtered and then concentrated using a water bath with shaker at 56°C in clean, pre-weighed glass beakers. The obtained semisolid residue (yield) was weighed and re-constituted in a measured amount of isotonic saline (0.85% NaCl, w/v). Yield percentage was calculated as: (extracted residue weight / original seed weight) × 100.

For the *in vitro* study, the reconstituted extract concentration was first adjusted to 1 mg/mL, and then serially diluted in isosaline to obtain 512, 256, 128, 64, 32, 16, 8, and 4 μg/mL solutions. For the *in vivo* study, the reconstituted extract concentration was adjusted to 20 and 40 mg/mL (for the doses of 100 and 200 mg/kg, respectively; a rat weighing 200 g body weight received 1 mL of an extract by gastric tube). Extracts for phytochemical testing were prepared appropriately according to the test applied to the group as mentioned below.

### *In vivo* hematinic procedure

#### Animals and experimental design

A total of 30 male albino rats weighing 200 g on average were used. The rats were housed hygienically in metabolic cages kept in a conditioned room (12-h light-dark cycles, temperature 25^o^C) and clean water and a balanced diet were provided *ad libitum*. After acclimatization for a week, rats were divided into 5 groups (6 rats each) in 5 separate suitable-sized cages and subjected to different treatments. Control group (I) was injected and orally administered only with the vehicles of phenylhydrazine and *B. vulgaris* extract (BVE), respectively, at the corresponding time-points. Diseased group (II) was injected with 20 mg/kg phenylhydrazine, intraperitoneally, for two successive days (days 1 and 2), and orally administered with only the vehicle of BVE at the specified time points. Standard group (III) was injected with phenylhydrazine in the same manner as group II, and orally administered with ferrous ascorbate-folic acid combination in saline (13.5+0.135 mg/kg, daily for 12 days, using a stomach tube) as standard anti-anemics. Treated groups (IV and V) were injected with phenylhydrazine in the same manner as group II, and orally administered BVE in saline (100 and 200 mg/kg, respectively, daily for 12 days, using a stomach tube) as the tested anti-anemic. All procedures were ethical for animals and performed with merciful and humane manner according to the recommendations of our Institutional Animal Care and Use Committee (IACUC), Benha University.

#### Sampling

Along the course of the experimental period, blood samples were obtained on days 0, 4, 8, and 12 from the medial canthus venous plexus under light ether anesthesia. Each blood sample was collected into plain and EDTA-containing tubes. The blood fraction in the EDTA-tube was gently mixed and used directly for blood analysis while the fraction in the plain tube was left to coagulate, centrifuged at 605 *g* for 5 min at room temperature, and serum was collected in labelled Eppendorf tubes and used for determination of erythropoietin (EPO) and malondialdehyde (MDA).

#### Hemogram

To evaluate the hematinic potential of BVE, the following hematological parameters were automatically evaluated by an auto-hematology analyzer (Mindray^®^, Model BC-2800Vet, China). Erythrocytic parameters included RBC count, hematocrit value (HCT), hemoglobin concentration (HGB), mean corpuscular volume (MCV), mean corpuscular hemoglobin (MCH), and mean corpuscular hemoglobin concentration (MCHC). Leukocytic parameters included total white blood cell (WBC), lymphocyte, granulocyte, monocyte, and platelet (PLT) counts (18).

#### Erythropoietin assay

Erythropoietin levels were determined in serum samples using rat EPO ELISA kit purchased from HexaBiogen^®^ (Tunisia, for MyBioSource, Inc., USA). The assay employs the quantitative sandwich enzyme immunoassay technique that was followed according to the instructions of the manufacturer. The color intensity was measured at 450 nm using an ELISA microplate reader.

#### MDA assay

MDA concentrations in serum were measured as an indicator for the extent of erythrocytic membrane lipid peroxidation, following the original method (19) with some modifications. This was performed using thiobarbituric acid solution, which reacts with MDA giving a pinkish color. Briefly, 0.5 mL of serum, 0.5 mL isosaline, and 0.5 mL 25% trichloroacetic acid were mixed in glass tubes and centrifuged at 605 *g* for 20 min at room temperature. An aliquot of 1 mL of protein-free supernatant was mixed with 0.25 mL and 0.5% thiobarbituric acid and heated at 95°C for 1 h. After cooling, the intensity of pink color was determined at 532 nm. The MDA concentration was calculated according to the following formula:

$$\mathrm{MDA(µM)}=\frac{A_{sample}}{l\times є}\times DF$$

where: *l* = light path = 1 cm, ε = molar absorptivity factor = 1.56 X 10^5^ M^-1^ cm^-1^, and DF = dilution factor = 21.

### *In vitro* DPPH assay

This assay was according to Blois (20) and Manzocco, et al. (21). The test principle depends on that the molecule 2,2′-diphenyl-1-picryl-hydrazyl (DPPH^-^) is categorized as a stable free radical species by rule of the delocalization of the spare electron over the molecule components, so that the molecule cannot dimerize. Such electron delocalization gives the molecule deep violet color when in ethanol or methanol as solution, with an absorption band measured at 517 nm. When a solution of DPPH^-^ is mixed with another solution that can donate a hydrogen atom, it gives rise to the reduced form of DPPH with the loss of this violet color. An aliquot of the extract test sample (200 μL) at a specific concentration (4∼512 μg/mL) was added to 2 mL of DPPH^-^ solution (0.5 mM in methanol) in a clean and dry covered test tube. A blank control and standard tubes were prepared by substituting the test sample solution with isosaline and ascorbic acid (2 mM in saline), respectively. After 30 min of incubation at room temperature, the absorbance was measured at 517 nm. The percentage of the DPPH^-^ free radical scavenging is calculated using the following equation: Antioxidant % = [(A_blank_ - A_sample_) / (A_blank_ - A_standard_)] × 100. The decrease of the degree of the violet color indicates higher antioxidant activity

#### Phytochemical analysis

Phytochemical detection tests for presence of phenols and flavonoids in foliage of *B. vulgaris* were carried out as described previously (22). The tests were performed as triplicates and given marks from (-) to (+++) according to the strength of the color or precipitate that appeared.

### Statistical analysis

Data are reported as means±SE of the mean of three (*in vitro*) or six (*in vivo*) separate observations. *In vivo* observations were compared using ANOVA followed by least significant difference (LSD) as the *post hoc* test where P was set at 0.05. *In vitro* observations were calculated as percent of the activity of the corresponding standard. The IC50 value of the BVE was calculated from the logarithms of the used concentration range (4∼512 μg/mL). All statistics and graphing were done using the computer program GraphPad Prism^®^ version 6 (GraphPad Inc., USA).

## Results

The yield percentage of the dried *B. vulgaris* leaf and stalk when macerated in hydro-ethanol (70:30, v/v) was 21.85%.

Data of the present study showed that the daily administration of BVE significantly and dose dependently affected both the erythrocyte and leukocyte parameters positively compared to the diseased group (P<0.05). BVE at small and large doses (groups IV and V, respectively) significantly restored RBCs count, HC, HGB, MCV, and MCHC values in dose- and time-dependent manners (Table 1).

**Table 1.** Erythrogram after intraperitoneal injections of phenylhydrazine (20 mg/kg bw, for two consecutive days) and oral administration of *Beta vulgaris* leaf and stalk extract (BVE-LD, BVE-SD, 100 and 200 mg/kg bw, for 12 consecutive days) on erythrocytic parameters of rats compared to those after the standard ferrous ascorbate+folate (13.5+0.135 mg/kg bw, for 12 consecutive days) and normal control (saline).

Days	Parameters	Groups				
		Control	Diseased	Standard	BVE-SD	BVE-LD
0	RBC (10^12^/L)	7.47±0.26	7.37±0.26	7.50±0.29	7.33±0.36	7.17±0.33
	HCT (%)	43.33±2.03	43.83±2.32	44.67±2.35	44.61±3.21	45.10±2.18
	HGB (g/dL)	13.67±1.36	13.57±1.28	13.70±1.30	13.50±1.33	13.83±1.30
	MCV (*f*L)	60.01±2.89	57.33±2.33	62.01±3.22	62.33±2.73	60.33±2.91
	MCH (pg)	18.03±1.44	17.71±1.35	18.04±1.29	17.53±1.19	17.97±1.39
	MCHC (%)	31.18±2.32	31.19±2.20	31.02±2.14	30.55±1.97	31.01±2.08
4	RBC (10^12^/L)	7.43±0.27	3.63±0.07^+^	6.13±0.18*	5.53±0.12*	5.70±0.15*
	HCT (%)	43.43±1.85	32.35±2.9^+^	38.66±2.75*	35.93±3.07	37.21±2.83*
	HGB (g/dL)	13.70±1.42	6.77±0.15^+^	9.90±0.49*	8.47±0.29*	8.71±0.27*
	MCV (*f*L)	58.00±2.65	88.3±3.22^+^	63.33±2.41*	64.3±6.10*	65±5.78*
	MCH (pg)	18.13±1.16	18.55±8.7	16.22±1.29	15.37±1.39	15.46±1.36
	MCHC (%)	31.21±2.42	20.97±1.7^+^	25.67±1.72*	23.59±1.73	23.53±1.79
8	RBC (10^12^/L)	7.47±0.15	4.80±0.15^+^	6.47±0.15*	6.01±0.16*	6.27±0.19*
	HCT (%)	43.73±1.84	34.23±2.7^+^	40.17±2.45*	38.4±2.66	40.91±2.93*
	HGB (g/dL)	14.13±1.39	8.50±0.29^+^	10.83±0.49*	9.61±0.23	10.11±0.26*
	MCV (*f*L)	55.67±2.97	72.7±5.61^+^	62.10±2.89*	63.76±2.2*	64.1±2.89*
	MCH (pg)	18.72±1.36	17.67±1.07	17.03±1.16	16.10±1.06	16.23±1.13
	MCHC (%)	32.14±1.42	24.74±1.6^+^	26.70±1.79	25.07±1.79	24.82±1.80
12	RBC (10^12^/L)	7.60±0.21	5.83±0.18	6.81±0.32	6.43± 0.13*	6.70±0.12
	HCT (%)	43.87±1.96	38.53±2.61	41.43±2.46	41.16±2. 17	44.26±2.53
	HGB (g/dL)	14.13±1.40	10.56±0.31^+^	11.77±0.15	10.71±0.28	11.10±0.27
	MCV (*f*L)	54.33±2.33	67.33±2.03^+^	60.26±2.87	63.5±2.17	64.43 ±3.38
	MCH (pg)	18.58±1.12	18.05±1.14	17.2±1.07	16.72±1.13	16.76±1.18
	MCHC (%)	32.27±1.33	26.83±1.95	27.97±1.32	26.12±1.64	25.46±1.94

Data are reported as means±SE for n=6. RBC: red blood cells; HTC: hematocrit value; HGB: hemoglobin concentration; MCV: mean corpuscular volume; MCH: mean corpuscular hemoglobin; MCHC: mean corpuscular hemoglobin concentration. ^+^P*<*0.05 compared to control; *P*<*0.05 compared to diseased (ANOVA).

Both tested doses of BVE had significant (P<0.05) normalizing effects on most leukocytic parameters including lymphocytes and granulocytes compared to the diseased group (Table 2).

**Table 2.** Leukogram and platelet count after intraperitoneal injections of phenylhydrazine (20 mg/kg bw, for two consecutive days) and oral administration of *Beta vulgaris* leaf and stalk extract (BVE-LD, BVE-SD, 100 and 200 mg/kg b wt., for 12 consecutive days), compared to those after the standard ferrous ascorbate+folate (13.5+0.135 mg/kg bw, for 12 consecutive days) and normal control (saline).

Days	Parameters	Groups				
		Control	Diseased	Standard	BVE-SD	BVE-LD
0	WBC (10^9^/L)	17.47±1.21	17.37±1.28	17.56±1.39	17.07±1.28	17.30±1.35
	Lymphocytes (%)	69.67±3.18	69.03±3.79	69.47±2.95	70.31±3.21	70.10±2.93
	Mid-sized (%)	0.73±0.36	0.81±0.36	0.70±0.32	0.74±0.34	0.74±0.32
	Gran (%)	12.26±1.10	11.33±0.73	11.71±0.92	12.03±1.13	12.53±1.21
	PLT (10^9^/L)	455.7±7.5	453.1±9.9	452.6±8.9	461.2±9.4	466.7±8.63
4	WBC (10^9^/L)	17.51±1.39	25.63±2.88^+^	19.13±1.24*	22.03±2.12*	20.13±1.85*
	Lymphocytes (%)	69.43±2.86	80.35±2.76^+^	74.69±2.75*	78.43±2.07	75.96±2.30*
	Mid-sized (%)	0.74±0.41	1.02±0.15	0.74±0.39*	0.75±0.31*	0.77±0.34*
	Gran (%)	12.30±1.05	20.31±1.36^+^	15.03±0.91*	18.34±0.78	17.06±0.98*
	PLT (10^9^/L)	459.3±6.96	479.34±8.09	464.35±7.63	465.5±7.85	464.2±6.53
8	WBC (10^9^/L)	17.53±1.35	23.08±2.07^+^	18.07±1.30*	20.01±1.12*	17.91±1.19*
	Lymphocytes (%)	70.33±2.84	77.23±2.03^+^	73.17±2.09*	76.04±2.06	73.93±2.10*
	Mid-sized (%)	0.78±1.39	1.30±0.19	0.83±0.34*	0.77±0.23	0.80±0.25*
	Gran (%)	12.17±0.97	18.07±1.01^+^	13.71±1.09*	17.10±0.78	15.17±0.91*
	PLT (10^9^/L)	458.67±7.3	473.4±7.8	459.6±8.5	465.1±8.5	462.3±7.8
12	WBC (10^9^/L)	17.60±1.42	20.03±2.29	17.56±1.18*	18.53± 1.63*	17.70±1.72*
	Lymphocytes (%)	70.61±2.76	75.11±2.71	72.34±2.06	74.10±2.10	72.16±2.73
	Mid-sized (%)	0.79±0.23	0.98±0.40	0.78±0.24	0.78±0.23	0.83±0.23
	Gran (%)	12.37±0.93	17.33±1.03^+^	13.06±0.76*	16.06±0.88	13.46±0.84*
	PLT (10^9^/L)	457.2±7.8	470.7±7.5	454.3±12.3	461.7±8.6	455.8±9.6

Data are reported as means±SE; n=6. WBC: white blood cells; Mid-sized: (monocytes); Gran: (granulocytes: neutrophils, eosinophils, basophils); PLT: platelet counts. ^+^P*<*0.05 compared to control; *P*<*0.05 compared to diseased (ANOVA).

**Figure 2.**
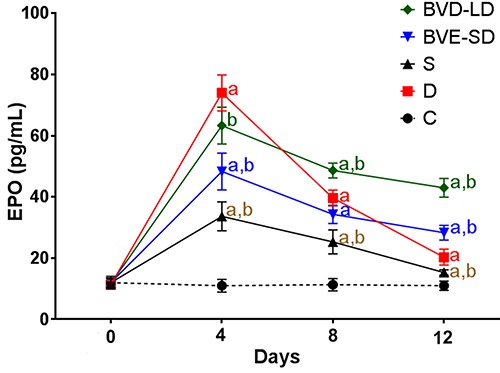
Erythropoietin (EPO) serum concentrations (pg/mL) after intraperitoneal injections of phenylhydrazine (20 mg/kg bw, for two consecutive days) and oral administration of *Beta vulgaris* leaf and stalk extract (BVE-LD and BVE-SD, 100 and 200 mg/kg bw, for 12 consecutive days) compared to standard (S) ferrous ascorbate+folate (13.5+0.135 mg/kg bw, for 12 consecutive days) and normal control (C). D: diseased control. Data are reported as mean±SE for n=6, P*<*0.05: a: compared to control; b: compared to diseased (ANOVA).

**Figure 3.**
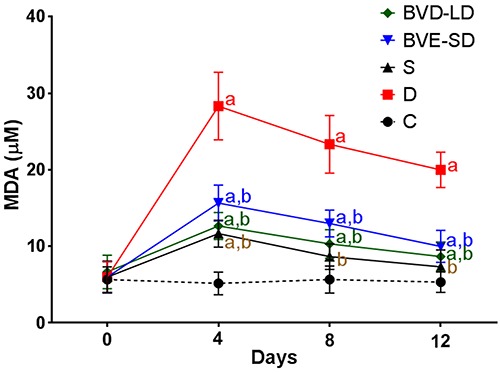
Malondialdehyde (MDA) serum concentrations after intraperitoneal injections of phenylhydrazine (20 mg/kg bw, for two consecutive days) and oral administration of *Beta vulgaris* leaf and stalk extract (BVE-LD and BVE-SD, 100 and 200 mg/kg bw, for 12 consecutive days) compared to those after the standard (S) ferrous ascorbate+folate (13.5+0.135 mg/kg bw, for 12 consecutive days) and normal control (C). D: diseased control. Data are reported as means±SE for n=6. P*<*0.05: a: compared to control; b: compared to diseased (ANOVA).

Serum analysis assays revealed that BVE significantly (P<0.05) maintained higher EPO concentration than both control and diseased groups after the 4th day of the experiment. MDA levels were significantly (P<0.05) decreased compared to the diseased group after continuous administration of BVE for 12 days. Both increasing and decreasing effects of BVE on EPO and MDA, respectively, were dose- and time-dependent (Figure 2 and 3).

**Figure 4.**
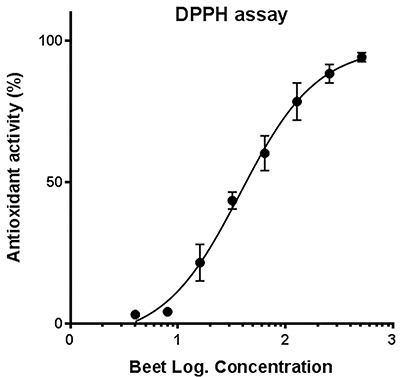
DPPH^-^ scavenging by different concentrations of *Beta vulgaris* leaf and stalk hydroethanolic extract (4∼512 μg/mL). Data are reported as means±SEM of triplicates.

Data of the present study revealed that BVE exhibited potent total antioxidant activity indicated by the DPPH assay. The effect was concentration-dependent with IC50 values of 37.91 μg/mL (Table 3 and Figure 4).

**Table 3.** *In vitro* antioxidant activity (% of 2 mM ascorbate as a standard) of *Beta vulgaris* leaf and stalk extract (4∼512 μg/mL) indicated by DPPH assay.

Concentration	Antioxidant activity (%)
4 μg/mL	3.23±0.29
8 μg/mL	4.26±0.52
16 μg/mL	21.61±3.72
32 μg/mL	43.58±1.72
64 μg/mL	60.31±1.89
128 μg/mL	78.58±1.89
256 μg/mL	88.47±1.89
512 μg/mL	94.26±0.93

Data are reported as means±SE of 3 replicates.

Phytochemical analysis revealed the presence of tannins, gallic acid, and flavonoids indicated by strong reactivity of BVE with the detecting reagents (Table 4).

**Table 4.** Phytochemical analysis results of the leaf and stalk extract of *Beta vulgaris*.

Active principle group	Test	Result
Tannin	Gelatin	+++
	Lead acetate	+++
	Phenazone	+++
	FeCl_3_ test	+++
Phlobatannin	Hydrochloric acid test	++
Gallic acid	Vanilin test	++
Flavonoids	Shinoda's test	+++
	Wilson's	+++
	Lead acetate	+++
	Alkaline reagent	+++

## Discussion

Nature is and will be an endless source for remedial preparations for different disease conditions that may be life threatening to all beings. Anemia is a multifactorial disease condition from which a large population suffers, especially females of developing countries. Search for novel anti-anemics is, therefore, considered an attractive research subject by many scientists, including pharmacologists and pathologists.

Finding an anti-anemic preparation necessitates a suitable experimental anemia model. The phenylhydrazine model is an acute model for hemolytic anemia that lasts from 8 to 12 days and is considered a good, rapid tool for an investigational study. The model induces anemia within 4 days after injection of phenylhydrazine, comprising erythrocytopenia (about 50%), lowered Hb (about 60%), and reduced HCT. The model also comprises reticulocytosis (up to 475%) on day 7 after injection accompanied by higher EPO levels as a body defensive reflex action against the induced anemia. Higher indices of MCV and leukocytosis (neutrophilia and lymphocytosis) are also established findings of the model. The mechanistic action by which phenylhydrazine induces such anemic findings is mainly attributed to its oxidative stress on erythrocytes and lipid peroxidation of its membranes indicated by higher MDA serum levels (23,24).

The beet is a very attractive plant by virtue of its red-purple coloration that gives a feeling of compensating lost blood in folk medicine. Moreover, on the scientific scale, some of its pharmacological effects have been proved as mentioned in the introduction section. In the current study, it was hypothesized that BVE, based on its antioxidant potential, may guard against development of anemia caused by oxidative stress drugs and hemolyzing agents. Therefore, we have designed a challenge experiment between *B. vulgaris* extract and phenylhydrazine in rats aiming at giving scientific evidence for the possible hematinic action of the extract.

The hemogram of the present study showed a significant reduction in RBC count, HGB concentration, and HCT values in rats on days 4 and 8 post-injection of phenylhydrazine. By the end of the experimental course (day 12), the hematological effects of phenylhydrazine declined or were self-limited by the body reflexive mechanisms. Intervention with BVE significantly protected against the deteriorating effects of phenylhydrazine on the levels of RBCs, HGB, and HCT at each time-point of analysis, especially on day 4, the most critical time-point. Among erythrocytic indices, MCV was significantly decreased (improved) and MCHC was insignificantly increased by BVE compared to those of the diseased control. On the other hand, MCH did not show a difference, which is a logic attribute as it is a ratio of HGB and RBCs that remain parallel (direct relationship) in this type of anemia. Reticulocytes were speculated to be lower than that of diseased group but higher than the control group relaying on EPO data.

Table 3 shows the normalizing effects of BVE on the abnormally induced leukogram by phenylhydrazine. Neutrophilia and lymphocytosis induced by the stress of phenylhydrazine and reflexive erythropoietin release were almost normalized at each time-point. Such normalizing effect may be attributed to the impediment the oxidative stress and modulation of EPO release. This is supported by the decreased MDA concentration in the extract-treated groups and *in vitro* antioxidant potential of BVE.

As shown in Figure 2, phenylhydrazine injection was associated with an elevated level of serum erythropoietin, with a peak on day 4. Although intervention with BVE limited the maximal elevation of erythropoietin (on day 4), afterwards, the extract maintained relatively high levels of erythropoietin (days 8 and 12). This may indicate that BVE has EPO stimulating effects on the kidney and other tissues, unlike phenylhydrazine that is associated with reflex or indirect EPO release after its anemic effect.

The present findings may be consistent with the favorable effects of *B. vulgaris* that have been reported previously using different extract types and parts and in other disease models. For instance, Jaiswal et al. (25) showed that treatment of anemic rats with extracts of the plant root prepared by hot percolation in a Soxhlet apparatus significantly reversed the decrements of RBCs count and HGB concentration. More recently, Al-aboud (26) found that taking eight grams of beetroot for 20 days by female volunteers resulted in mild increase in hemoglobin, ferritin, and serum iron levels.

One of the major mechanisms by which phenylhydrazine induces hemolytic anemia is by induction of oxidative stress and this was confirmed by finding that MDA serum levels were higher compared to the control group along the experimental course, especially on the day 4. However, concurrent administration of BVE with phenylhydrazine significantly protected against the oxidative effect on erythrocytic membranes in all tested serum samples, confirmed *in vitro* by DPPH assay, where BVE exhibited a potent, concentration-dependent, antioxidant activity with IC50 of 37.91 μg/mL.

The antioxidant potential of BVE, both *in vivo* (MDA decrement) and *in vitro* (DPPH^-^ clearance) could be explained by its rich content of flavonoids, tannins, and gallic acid, in accordance with previous findings of Babu and Gowri (27) who reported that simultaneous intraperitoneal administration of beet methanolic extracts (100 and 200 mg·kg^-1^·day^-1^) with CCl_4_ (1 mL/kg) to rats every other day for two weeks prevented membrane lipid alteration in RBCs induced by oxidative stress. Our antioxidant and phytochemical data are also in accordance with Kähkönen et al. (28) who evaluated the antioxidative activities of 92 phenolic extracts from edible and nonedible plant materials (including beetroot peel) using autoxidation of methyl linoleate.

In conclusion, the extract of *B. vulgaris* leaf and stalk showed strong hematinic and anti-anemic potentials based on its antioxidant principles. The extract, thus, could be a good and promising natural source for hematinic and antioxidant pharmaceutical preparations.
